# Methodological Research Practices for the Adaptation of Measuring Instruments in Health Care: Protocol for a Scoping Review

**DOI:** 10.2196/86949

**Published:** 2026-03-27

**Authors:** Ninive Pita Gomes de Oliveira, Rosilene Alves Ferreira, Andressa Aline Bernardo Bueno, Flávia Giron Camerini, Danielle de Mendonça Henrique, Cristiane Helena Gallasch, Cintia Silva Fassarella

**Affiliations:** 1 Faculdade de Enfermagem, Universidade do Estado do Rio de Janeiro, Rua São Francisco Xavier, 524, Maracanã, Rio de Janeiro, 20550-900, Brazil, 55 23340000

**Keywords:** psychometrics, transcultural adaptation studies, health, research instruments, nursing

## Abstract

**Background:**

The identification of the methodological path followed by researchers in psychometric studies can contribute to maintaining the transparency and rigor necessary for the study.

**Objective:**

This study aims to map the methodological practices of adaptation of health measurement instruments.

**Methods:**

The protocol is based on the method proposed by the Joanna Briggs Institute using the PRISMA-ScR (Preferred Reporting Items for Systematic Reviews and Meta-Analyses extension for Scoping Reviews). The following databases were consulted: MEDLINE, CINAHL, Web of Science, LILACS, the Cochrane Library, and Embase. The search for data in gray literature sources was carried out in the following databases: WorldCat and the Brazilian Portal of Scientific Publications and Open Access Data (O Portal Brasileiro de Publicações e Dados Científicos em Acesso Aberto [OASISBR]). The search strategy was based on the participants, concept, and context framework: participants (researchers in methodological practices), concept (studies of adaptation of measuring instruments), and context (health area). The research will consider studies on methodological practices adopted to conduct adaptation studies of health measurement instruments. The selection of studies, data extraction, and synthesis will be carried out by 2 independent reviewers.

**Results:**

Data collection was carried out in the months of June and October 2025. The results will be presented in a narrative format and with tables or diagrams. The results are being prepared for publication and should be published by March 2026.

**Conclusions:**

This study will identify the current status and challenges faced by researchers in carrying out adaptation studies of health measurement instruments. The findings will contribute to the development of future research that promotes better psychometric evidence and advances science in nursing.

## Introduction

Psychometrics is based on the theory of measurement in sciences to explain the sense that have the answers given by participants to a series of tasks, typically called items, and thus proposes techniques for measuring mental processes. In summary, psychometrics seeks to understand the meaning behind the answers given by the respondents, and from this dynamic, it is possible to develop tools to evaluate psychological aspects [1].

From this, it is possible to understand that studies aimed at the adaptation of measurement instruments are essentially psychometric studies, and these studies concern themselves with the problems related to the challenges of measuring human behavior, through a specific set of mathematical methods and models, in addition to providing essential aspects for conducting research in social and behavioral sciences [2-4].

The adaptation of a test, that is, an instrument, is a complete scientific process, and as a result, it is guided by the principles of the scientific method—the need to offer evidence of the adequacy of this linguistic transformation being the most prominent of all—not only in terms of language but also in terms of other psychometric characteristics [5].

Several categories of professionals have made efforts to apply the measurement instruments in their practices, allowing the evaluation and measurement of different behaviors and variables. Such behavior has also been seen in health and nursing professionals, who have been developing research aimed at creating or adapting instruments that can improve their professional practices [6-9].

However, it is essential that the conduct of these studies is anchored in a contemporary repertoire of recommendations, which can contribute to resolving the weaknesses inherent in the study and capable of reverberating and impacting positively the reality and designated purpose [10,11]. In this context, it is important to mention that the international scientific community recognizes the standards of the International Test Commission [10] as general guidelines for the process of transcultural adaptation, simultaneously between countries [10]. In 2017, a refinement of the original 1996 proposal was published, in which the guidelines were presented that establish general recommendations and guidelines for conducting studies of this nature and with this purpose [10].

In addition, the periodic updates proposed by the Standards for National and Psychological Test (2014 Edition) [12] are published and disseminated according to the need to conduct psychometric studies aimed at adapting the instruments based on scientific evidence. These updates aim to ensure the required transparency and methodological rigor, as well as to ensure that the tests meet scientifically established quality parameters [12,13].

It should be noted that there is no consensus on how to adapt or create an instrument for use in another cultural context. There are no recipes, manuals, or step-by-step procedures on how to do it. This procedure will depend on the characteristics of the instrument and the context of its application and the population to which it is intended. However, there is a consensus that the process of adaptation goes beyond mere translation. Moreover, validity cannot be guaranteed based on isolated evidence alone, as the validation process requires the accumulation of different relevant evidence to provide a solid scientific basis for interpretations of proposed scores [14,15].

In this sense, the identification of the methodological guidelines that guide these studies is a fundamental strategy for improving the evaluation of quality and safety in the process of adaptation of instruments, allowing the verification of methodological consistency, as well as the alignment between the tools produced for health practice and the theoretical and normative recommendations established by the experts of the method.

Therefore, conducting research through psychometric studies is justified, as it supports scientific advancement in the field, enables the measurement of sensitive and unique variables, and provides interpreted results and analyses that can inform decision-making. In addition, it is estimated that the identification of the practices conducted in these studies may, in the future, be able to guide researchers in the field to improve their practices and expand the contemporary knowledge recommended for studies with this scope.

A preliminary search was conducted on April 4, 2025, to identify other available reviews with a similar objective as that of this study. The survey included the Cochrane database of systematic reviews, the Joanna Briggs Institute’s Synthesis of Evidence, and the Open Science Framework repository, and no data were found that would prevent the referral of this research.

The research question of this protocol is as follows: What are the methodological practices of adaptation of measurement instruments in health? Thus, the objective is to map the methodological practices of adaptation of measurement instruments in health care.

## Methods

### Overview

The proposed protocol will be carried out according to the Joanna Briggs Institute (JBI) methodology for scoping reviews and the PRISMA-ScR (Preferred Reporting Items for Systematic Reviews and Meta-Analyses extension for Scoping Reviews) [16,17]. The protocol followed the PRISMA-P (Preferred Reporting Items for Systematic Reviews and Meta-Analyses Protocols) and was registered on the Open Science Framework[18].

### Eligibility Criteria

The eligibility criteria were developed according to the participants, concept, and context (PCC) framework, and these will be discussed in the following sections [16].

#### Participants

The participants in this review correspond to the researchers responsible for conducting adaptation studies of measuring instruments. Therefore, scientific productions that describe the methodological decisions, procedures, and steps adopted by researchers in the adaptation process are considered including, where applicable, translation, back-translation, synthesis, evaluation by a committee of experts, and pretest and psychometric analysis.

The adoption of the participants criterion is justified by the fact that, in scoping reviews, as recommended by the JBI [16], participants may correspond to different analytical units, not restricting themselves to individuals but including authors, studies, or processes. In this perspective, the focus on the researchers makes it possible to identify, describe, and analyze systematically the methodological conduct, the technical decisions, and the steps adopted in the processes of adaptation of measuring instruments [17,19].

#### Concept

For the concept portion of the framework, as a definition, adaptation studies of measurement instruments will be considered, with emphasis on methodological processes used for the transcultural or linguistic adaptation of instruments originally developed in other contexts. The review seeks to map, describe, and systematize the methodological steps adopted in these studies, as well as the theoretical and normative recommendations that guide such processes.

The justification for this criterion lies in the diversity of studies carried out in the field, as some of these studies use obsolete and outdated techniques and procedures that may not produce reliable and adequate evidence to support the adaptation process of instruments and may introduce benchmarking bias in clinical practice in health [20-22].

#### Context

In relation to the context of this protocol, the health area will be understood, covering different disciplinary fields, levels of care, and educational and research scenarios. Studies that involve the adaptation of measurement instruments intended for the evaluation of constructions related to human health, clinical practice, public health, health management, and scientific research will be considered, regardless of the target population or level of complexity of care, since inserted in the scope of health sciences.

The justification of the criterion lies in the possibility of intervention for more accurate diagnoses, effective treatments, and patient safety with the use of measuring instruments intended for the health care context. In addition, instruments intended for and applied in the health field allow the quantification of several sensitive variables, and they can assist in the assessment of health status and clinical decision-making [20].

### Search Strategy

On the basis of the terms used in the acronym PCC, a mapping of keywords was carried out to identify their descriptors in the controlled health area vocabularies, including Descriptors in Health Sciences, Medical Subject Headings (MeSH), NorthHolland Publishing Company (ELSEVIER), and CINAHL. The terms contained in the titles and abstracts of the relevant articles obtained and the descriptors used will be used to develop a complete search strategy. A librarian with expertise in the health care field developed a structured search strategy with keywords, descriptors, and their synonyms, adapted to each database. There will be a 3-step search strategy.

First, a limited initial search was carried out on June 26, 2025, in the MEDLINE via PubMed and in the CINAHL via EBSCo databases to identify the existence of material that would support this research (Table 1).

**Table 1.** Search strategies and preliminary results. Rio de Janeiro, RJ, Brazil, 2025^a^.

Database	Search strategy	Results, n (%)
MEDLINE	(“Psychometrics/instrumentation”[Majr] OR “Psychometrics/methods”[Majr] OR “Psychometrics/standards”[Majr]) AND (Validation Studies as Topic[Mesh] OR Adaptation[Title/Abstract] OR Validation[Title/Abstract]) AND (“Health Workforce”[Mesh] OR “Health Personnel”[Mesh] OR “Nursing”[Mesh])	302 (49.3)
CINAHL	(Psychometrics OR “Psychometrics/ST” OR “Psychometrics/MT”) AND MM (“Instrument Adaptation” OR “Instrument Validation” OR “Validation Studies”) AND TX(“Nursing Care” OR “Nursing Diagnosis” OR “Nursing context” OR “health context”)	294 (50.7)

a Source: adapted by the authors, 2025.

A second survey using all identified keywords and synonyms was conducted on October 24, 2025, in the following databases: MEDLINE via PubMed, CINAHL, Web of Science, LILACS via BVS, Embase, and the Cochrane Library (Table 2).

**Table 2.** Search strategies and preliminary results. Rio de Janeiro, RJ, Brazil, 2025^a^.

Database	Search strategy	Results, n (%)
MEDLINE	(“Psychometrics/instrumentation”[Majr] OR “Psychometrics/methods”[Majr] OR “Psychometrics/standards”[Majr]) AND (Validation Studies as Topic[Mesh] OR Adaptation[Title/Abstract] OR Validation[Title/Abstract]) AND (“Health Workforce”[Mesh] OR “Health Personnel”[Mesh] OR “Nursing”[Mesh])	326 (19.3)
CINAHL	(Psychometrics OR “Psychometrics/ST” OR “Psychometrics/MT”) AND MM (“Instrument Adaptation” OR “Instrument Validation” OR “Validation Studies”) AND TX(“Nursing Care” OR “Nursing Diagnosis” OR “Nursing context” OR “health context”)	316 (18.7)
Web of Science	(“Psychometrics” OR “psychometric assessment” OR “psychometric properties”) AND (“Validation Studies” OR “cross-cultural adaptation” OR “Construction and evaluation”) AND (“Health Workforce” OR “Health Personnel” OR “Nursing”)	296 (17.5)
LILACS	((“Psicometria/instrumentação” OR “Psicometria/métodos” OR “Psicometria/normas”) OR (“Propriedades psicométricas” OR “validação psicométrica” OR “Avaliação psicométrica” OR “psychometric properties” OR “psychometric validation” OR “Psicometric evaluation” OR “psychometric assessment” OR “Evaluación psicométrica”)) AND (mh:(“Estudos de Validação como Assunto”) OR “Validation Studies as Topic” OR “Adaptação cultural” OR “Adaptação transcultural” OR “cross-cultural adaptation” OR “Construção e avaliação” OR “Construction and evaluation” OR “Elaboración y evaluación”) AND (mh:(“Mão de Obra em Saúde” OR “Pessoal de Saúde” OR “Enfermagem”) OR “Health Workforce” OR “Fuerza Laboral en Salud” OR “Main-d’oeuvre en santé” OR “Health Personnel” OR “Personal de Salud” OR “Personnel de santé” OR Nursing OR Enfermería OR Soins)	543 (32.0)
Embase	(“psychometry”/exp/mj AND “devices”/de OR (“psychometry”/exp/mj AND “methodology”/de) OR (“psychometry”/exp/mj AND “standard”/de)) AND (“validation study”/exp OR “adaptation”:ti,ab,kw OR “validation”:ti,ab,kw) AND (“health workforce”/exp OR “health care personnel”/exp OR “nursing”/exp)	175 (10.3)
Cochrane Library	(“Psychometrics/instrumentation”[Majr] OR “Psychometrics/methods”[Majr] OR “Psychometrics/standards”[Majr]) AND (Validation Studies as Topic[Mesh] OR Adaptation[Title/Abstract] OR Validation[Title/Abstract]) AND (“Health Workforce”[Mesh] OR “Health Personnel”[Mesh] OR “Nursing”[Mesh])	37 (2.2)

a Source: adapted by the authors, 2025.

The research in the gray literature, available in Table 3, was carried out in the same period with the aim of broadening the identification of evidence and reducing publication bias. The WorldCat databases and the Brazilian Portal of Scientific Publications and Data in Open Access (O Portal Brasileiro de Publicações e Dados Científicos em Acesso Aberto [OASISBR]) were consulted, which collect theses, dissertations, technical reports, and other academic documents. The same descriptors and search strategies applied to bibliographic databases were used, with adaptations to the specificities of each platform.

**Table 3.** Strategies to search gray literature. Rio de Janeiro, RJ, Brazil, 2025^a^.

Database	Search strategy	Results, n (%)
WorldCat	“psychometrics properties” AND (adaptation OR construction) AND health	84 (17.5)
OASISBR	(“psychometrics properties” OR “propriedades psicométricas” AND (adaptation OR adaptação) AND Health NOT “revisão sistemática” NOT nutrição NOT desporto NOT (occupational OR ocupação)	397 (82.5)

a Source: adapted by the authors, 2025.

In the third step, the reference lists of articles included in the review will be evaluated for possible selection of additional articles. It should be noted that the review will consider studies in any language and without a time restriction, given that the recommendations for conducting research of this scope are part of a historical trajectory rooted in American Psychological Association guidelines [12], which have been disseminated and implemented in practice since 1950, more specifically since 1952.

### Types of Sources

Regarding the methodological approach, the review will include cross-cultural, linguistic, or contextual adaptations. It will include studies published in peer-reviewed journals, dissertations, theses, and technical reports detailing methodologies of adaptation and psychometric analysis because these documents provide detailed information on the methodological steps, technical decisions, and procedures adopted by the researchers.

To minimize the risk of double counting of evidence already summarized in reviews, each identified study will be individually checked for its original publication. Whenever a primary study is present in an earlier review, only the original publication will be included in the review. This ensures that each instrument adaptation is considered only once, preserving the integrity and accuracy of the mapping of methodological practices [10,12].

### Study Selection

After the search, all identified studies will be grouped and exported to Rayyan [23], and duplicates will be removed. The titles and abstracts will be analyzed by 2 independent reviewers, after training, to assess the studies’ eligibility in relation to the inclusion criteria initially defined. A screening pilot test will be conducted independently by both reviewers on an initial total of 25 titles and abstracts. The reviewers will discuss discrepancies and make changes to eligibility criteria and definitions if necessary. This pilot test will continue until at least 75% agreement is reached among the reviewers [16,24].

Complete studies that meet or potentially meet the inclusion criteria will be reviewed. The full text of the selected references will be evaluated according to the inclusion criteria by 2 independent reviewers. Any disagreements between the reviewers at each stage of the selection process will be resolved by consensus or by decision of a third reviewer. Eligible study references will be imported into the Mendeley reference manager. Any reasons for exclusion of studies will be recorded and reported in the scoping review. The research results will be reported in full in the final scoping review and presented in a flowchart.

### Data Extraction

Data will be extracted from studies included in the scoping review by 2 reviewers using a data extraction instrument adapted from the model proposed by the JBI [16] (Textbox 1). The data extracted will include specific details about the studies consulted, including information such as title, authors, year of publication, place of study, type of study, research object, research issue, study objectives, study population, sample size, study results, study procedures, study procedures, data analysis, and gaps and limitations of the study.

Textbox 1. **Data extraction instrument. Rio de Janeiro, RJ, Brazil, 2025.**

**Scoping review details**

**Title**

- Methodological research practices for adaptation of measuring instruments in the health field: protocol for a scoping review

**Objective**

- Map the methodological practices of adaptation of measurement instruments in health.

**Inclusion criteria**

- Participants: studies that are conducted by researchers on methodological practices, that is, steps conducted by researchers for adaptation studies of measurement instruments
- Concept: adaptation studies of measurement instruments available in the literature
- Context: health studies on the adaptation of measurement instruments to be used and applied in clinical practice

**Research issue**

- What are the methodological practices of adaptation of measurement instruments in health?

**Details extraction from selected studies**

- Title
- Authors
- Year of publication
- Place of study
- Type of study
- Object of research
- Research question of the study
- Objective of the study
- Population
- Size of sample
- Results of the authors
- Techniques adopted by the authors
- Procedures adopted by the authors
- Analysis of data
- Gaps or limitations
- Other variables related to the methodological practices adopted by researchers in psychometric studies of the adaptation of measurement instruments (if any)

Source: adapted from the Manual of the Joanna Briggs Institute, by authors.

The draft of the data extraction tool may be modified and revised if necessary, during the data extraction process for each included article, especially if variables related to the methodological practices adopted by researchers in psychometric studies of adaptation of measurement instruments are observed.

### Data Analysis and Presentation

The analysis for this scoping review will mainly adopt a content analysis, focusing on data extracted from the literature. As recommended by the JBI, the analysis will be predominantly descriptive and exploratory, without formal critical assessment of the quality of studies. This approach allows the results of the review to contribute directly to the understanding of methodological practices adopted in the adaptation of instruments, offering a comprehensive overview that supports future research and the implementation of good practices in health care [16].

The extracted data will be presented in tables or diagrams format, aligned with the research objective and issue of the scoping review. A descriptive summary will accompany the tabulated results and/or diagrams, providing context on how these data relate to the purpose of the review.

### Ethical Considerations

This review does not require approval from an ethics committee because it does not involve human participants.

### Dissemination

The results of the study will be disseminated through peer-reviewed publications, conference presentations, and targeted knowledge sharing sessions with relevant stakeholders.

It is believed that this synthesis of knowledge can offer valuable contributions to the academy and practice of nursing, as well as identifying gaps, enabling new paths for applied research, and pointing out transformations in the practice of studies with this scope and that impact on the application of tools that will be used in nursing practice.

## Results

Data collection was carried out in the months of June and October 2025. The results will be presented in a descriptive way, through a narrative synthesis, complemented by tables and/or diagrams, according to the nature of the extracted data. At the moment, the results are being organized and analyzed, with submission for publication by March 2026.

## Discussion

### Anticipated Findings

This scoping review aims to analyze and synthesize the existing literature on methodological practices adopted by researchers in carrying out studies on the adaptation of measuring instruments in the health care field.

To our knowledge, there is a lack of reviews on how the process of adapting measurement instruments occurs, which has resulted in numerous instruments being introduced and used in clinical practice, often with weak, incomplete, or insufficiently satisfactory methodological steps that do not meet the recommended standards for such studies. The use of instruments in clinical practice that do not present robust psychometric evidence may impact decision-making when it is based on the results of the instrument used.

Furthermore, the strength of this review lies in its ability to identify current gaps and possible weaknesses in studies on this topic, and above all, to provide a foundation for the development of future investigations that can deepen knowledge in the area and support the design and execution of measurement instrument adaptation studies based on contemporary methodological recommendations [9,11], directly contributing to the presentation of adequate and reliable psychometric evidence for each study design and phenomenon investigated.

### Limitations of the Study

Although the scoping review conducted according to the guidelines of the JBI is appropriate for the broad and systematic mapping of the literature, this methodological design has inherent limitations. The inclusion of studies with different methodological designs, contexts, and analytical approaches may have introduced heterogeneity in the data, limiting the depth of synthesis and comparison between results.

The search strategy, although structured and reproducible according to the JBI guidelines, was restricted to selected databases, which may have resulted in the exclusion of relevant productions not indexed or published in other languages. Finally, considering the exploratory nature of scoping reviews, the results obtained reflect the panorama of the literature available until the search period, not including studies published later, especially in areas of rapid scientific evolution.

### Conclusions

We hope that this study can contribute to building a discussion focused on the practice of researchers and the dissemination of results to multiple stakeholders, including nurses, health professionals, health managers, and certainly the populations for whom the measurement instrument is intended.

To maintain the relevance and significance of this context for clinical practice, it is believed that investigations with this type of scope should ensure methodological rigor supported by contemporary recommendations, in addition to ensuring that their decisions are documented so that it is possible to assess the credibility of the process that was followed, as well as assisting in the planning and execution of research of this type. Finally, this study will serve as a basis to equip researchers in the future to make decisions about the methodological framework to be used during their cross-cultural adaptation studies.

## Abbreviations

JBI: Joanna Briggs Institute
MeSH: Medical Subject Headings
PCC: participants, concept, and context
PRISMA-P: Preferred Reporting Items for Systematic Reviews and Meta-Analyses Protocols
PRISMA-ScR: Preferred Reporting Items for Systematic Reviews and Meta-Analyses extension for Scoping Reviews
